# Protective mechanism of *APOE3‐*Christchurch in Alzheimer’s disease

**DOI:** 10.1002/alz.093237

**Published:** 2025-01-09

**Authors:** Ana‐Caroline Raulin, Cynthia Linares, Alla Alnobani, Bhaskar Roy, Ailsa Crow, Sydney V. Doss, Lindsey A Kuchenbecker, Maxwell V Dacquel, Celeste M. Karch, Yuka A Martens, Chia‐Chen Liu, Guojun Bu, Takahisa Kanekiyo

**Affiliations:** ^1^ Mayo Clinic, Jacksonville, FL USA; ^2^ Department of Psychiatry, Washington University in St. Louis School of Medicine, St. Louis, MO USA

## Abstract

**Background:**

Homozygosity for the rare *APOE3*‐Christchurch (*APOE3Ch*) variant, encoding for apoE3‐R136S (apoE3‐Ch), was linked to resistance against an aggressive form of familial Alzheimer’s disease (AD). Carrying two copies of *APOE3Ch* was sufficient to delay autosomal AD onset by 30 years. This remarkable protective effect makes it a strong candidate for uncovering new therapies against AD. Thus, we aim to explore the protective mechanisms of *APOE3Ch* against AD onset, to inform therapy.

**Methods:**

We used both amyloid mouse models and human induced‐pluripotent stem cells (iPSC) models to address this aim. We examined whether astrocytic expression of apoE3‐Ch through an AAV‐mediated approach can mitigate AD‐related pathology and toxicity in early (3.5 months old) and late (8 months old) amyloid pathology in 5xFAD mice. We also used isogenic iPSC lines carrying the *APOE3Ch* variant that were generated from *APOE3* parental lines using CRISPR/Cas9 technology. Biochemical and biophysical properties of both recombinant and native apoE3‐Ch lipoprotein particles secreted by iPSC‐derived astrocytes have been investigated using different techniques including heparin affinity chromatography and size‐exclusion chromatography.

**Results:**

We investigated the impact of astrocytic apoE3‐Ch on amyloid pathology and toxicity in 3.5‐month‐old and 8‐month‐old 5xFAD mice. We confirmed successful AAV‐mediated overexpression of apoE3 and apoE3‐Ch in astrocytes. We found apoE3‐Ch specific changes in protein levels and solubility. Astrocytic expression of apoE3‐Ch reduced soluble Aβ oligomer levels and ameliorated ER stress response compared to apoE3 in the 8‐month‐old 5xFAD mice. Furthermore, our findings showed altered biochemical properties of HEK cell‐derived apoE3‐Ch protein, including decreased heparin binding, altered receptor binding, and enhanced lipid accepting capacity. We also characterized *APOE3Ch* iPSC‐derived cell models, including iPSC‐derived astrocytes, neurons, and cerebral organoids, and found differences between *APOE3* and *APOE3Ch* at the functional level in these iPSC models.

**Conclusions:**

The proposed work and the developed methods to study apoE3‐Ch promises to provide new insights into the possible roles of this rare, mutated protein in protecting against AD, offering new therapeutic avenues for AD treatment.

